# Preconditioning of mesenchymal stromal cells with low-intensity ultrasound: influence on chondrogenesis and directed SOX9 signaling pathways

**DOI:** 10.1186/s13287-019-1532-2

**Published:** 2020-01-03

**Authors:** Neety Sahu, Gaurav Budhiraja, Anuradha Subramanian

**Affiliations:** 1 Department of Chemical and Biomolecular Engineering, University of Nebraska-Lincoln, Lincoln, NE 68588-0643 USA; 2 Present Address: Department of Orthopaedic Surgery, School of Medicine, Stanford University, Stanford, 94304 USA; 3 Department of Chemical and Materials Engineering, The University of Alabama in Huntsville, Huntsville, AL 35899 USA

## Abstract

**Background:**

Continuous low-intensity ultrasound (cLIUS) facilitates the chondrogenic differentiation of human mesenchymal stromal cells (MSCs) in the absence of exogenously added transforming growth factor-beta (TGFβ) by upregulating the expression of transcription factor SOX9, a master regulator of chondrogenesis. The present study evaluated the molecular events associated with the signaling pathways impacting SOX9 gene and protein expression under cLIUS.

**Methods:**

Human bone marrow-derived MSCs were exposed to cLIUS stimulation at 14 kPa (5 MHz, 2.5 Vpp) for 5 min. The gene and protein expression of SOX9 was evaluated. The specificity of *SOX9* upregulation under cLIUS was determined by treating the MSCs with small molecule inhibitors of select signaling molecules, followed by cLIUS treatment. Signaling events regulating *SOX9* expression under cLIUS were analyzed by gene expression, immunofluorescence staining, and western blotting.

**Results:**

cLIUS upregulated the gene expression of *SOX9* and enhanced the nuclear localization of SOX9 protein when compared to non-cLIUS-stimulated control. cLIUS was noted to enhance the phosphorylation of the signaling molecule ERK1/2. Inhibition of MEK/ERK1/2 by PD98059 resulted in the effective abrogation of cLIUS-induced *SOX9* expression, indicating that cLIUS-induced *SOX9* upregulation was dependent on the phosphorylation of ERK1/2. Inhibition of integrin and TRPV4, the upstream cell-surface effectors of ERK1/2, did not inhibit the phosphorylation of ERK1/2 and therefore did not abrogate cLIUS-induced *SOX9* expression, thereby suggesting the involvement of other mechanoreceptors. Consequently, the effect of cLIUS on the actin cytoskeleton, a mechanosensitive receptor regulating *SOX9*, was evaluated. Diffused and disrupted actin fibers observed in MSCs under cLIUS closely resembled actin disruption by treatment with cytoskeletal drug Y27632, which is known to increase the gene expression of *SOX9*. The upregulation of *SOX9* under cLIUS was, therefore, related to cLIUS-induced actin reorganization. *SOX9* upregulation induced by actin reorganization was also found to be dependent on the phosphorylation of ERK1/2.

**Conclusions:**

Collectively, preconditioning of MSCs by cLIUS resulted in the nuclear localization of SOX9, phosphorylation of ERK1/2 and disruption of actin filaments, and the expression of *SOX9* was dependent on the phosphorylation of ERK1/2 under cLIUS.

**Electronic supplementary material:**

The online version of this article (10.1186/s13287-019-1532-2) contains supplementary material, which is available to authorized users.

## Background

As cartilage does not have the innate potential to regenerate, lesions frequently result in large-scale degenerative changes and osteoarthritis (OA) [1, 2]. The clinical outcomes of current strategies of cartilage repair autologous chondrocyte implantation (ACI) or matrix-assisted autologous chondrocyte implantation (MACI) are compromised by the phenotypic instability of expanded autologous chondrocytes ex vivo [3, 4] that leads to graft hypertrophy [5] and the formation of a mechanically inferior tissue in vivo. Therefore, regenerative approaches that employ progenitor cells such as mesenchymal stromal cells (MSCs) to improve cartilage repair outcomes are of interest.

Taking cues from the in vivo regulation of MSC chondrogenesis, current in vitro protocols include select growth factors (i.e., TGFβ) for differentiation of MSCs [6]. However, long-term conditioning of MSCs with TGFβ induces hypertrophy [5, 7] and calcification [8] upon terminal differentiation, leading to endochondral ossification instead of hyaline cartilage formation. Thus, chondroinductive protocols that do not rely on growth factors are of interest for the eventual development of ex vivo differentiation protocols for ACI and in situ repair strategies like microfracture.

Previously, a variety of biophysical stimuli, including mechanical stimulation, have been extensively studied in directing the differentiation of MSCs both in the absence and presence of growth factors [9–15]. Synergistic application of TGFβ with biomechanical forces yielded superior chondrogenic differentiation of MSCs in vitro, as evidenced by elevated expression of chondrocyte markers (Collagen II, SOX9, and aggrecan) [13, 14, 16]. However, as the mechanical stimulus was applied concurrently with TGFβ, the chondroinductive potential of the mechanical stimulus alone becomes indiscernible. Therefore, studies that critically examine MSC chondrogenesis in the absence of exogenously added growth factors are of significance.

In that regard, electrical stimulation and dynamic compressive loading have been documented to induce in vitro MSC chondrogenesis without the assistance of growth factors, as measured by the increased expression of chondrocyte markers, biochemical content, and mechanical stiffness over controls [12, 17–19], albeit the outcomes were inferior when compared to TGFβ-preconditioning [20–22]. Therefore, alternative methods of mechanical stimulation, including low-intensity ultrasound (LIUS), were explored for preconditioning MSCs toward a chondrogenic phenotype [23–25].

Low-intensity ultrasound (0.8 to 1.5 MHz, < 200 mW/cm^2^), applied as pulsed (pLIUS) or continuous (cLIUS) wave, has been documented to enhance the chondrocyte phenotype [26–28], improve cartilage repair [29, 30], and induce MSC chondrogenesis in vitro [25, 31] and in vivo [32], notably in the absence of exogenous chondroinductive biochemical factors [24, 33–35]. However, the growth factor-independent chondrogenic effect of pLIUS and cLIUS was either non-existent [31] or modest as evidenced by marginal increases in GAG and collagen content in 3D cultures of differentiated MSCs [34]. Differently from previous studies employing pLIUS or cLIUS at empirically derived frequencies (~ 1 MHz), theoretical modeling and experimental investigations conducted in our laboratory established that cLIUS couples more energy than pLIUS and cellular bioeffects are maximized at the cell resonant frequency of 5 MHz [36, 37]. For example, the long-term culture of MSC constructs receiving pLIUS stimulation at 1.5 MHz, a frequency outside the resonant bandwidth [36, 37], produced a substantially lower chondrogenic effect as evidenced by decreased biochemical content (GAG and collagen II) when compared to cLIUS stimulation at 5 MHz [34]. Additionally, the exposure of MSC constructs to cLIUS (5 MHz) for 8 weeks prevented the hypertrophic differentiation of MSCs by downregulating the expression of collagen X, a hypertrophic marker while sustaining the elevated expression of hyaline cartilage markers (SOX9 and collagen II) [38]. Taken together, cLIUS at 5 MHz was noted to be chondroinductive by acting as a stable inducer of chondrogenic differentiation in MSCs. Enhanced expression of the transcription factor SOX9, the master regulator of chondrogenesis [39–42], was observed in MSCs under pLIUS or cLIUS stimulation [24, 35, 38]; however, the underlying signaling events governing the upregulation of SOX9 are poorly understood.

In chondrocytes, a variety of signaling molecules are involved in the regulation of SOX9 [39, 41–45]. TGFβ induces chondrogenesis by regulating the phosphorylation of SOX9 through the SMAD (canonical) and p38 MAPK pathway (non-canonical) [43, 46, 47]. Mechanical stimulation by compressive loading was also reported to regulate the phosphorylation of SOX9 during MSC chondrogenesis by PKA [48] or by the autocrine TGFβ/SMAD [16] pathways. Despite the evidence of MSC chondrogenesis under mechanical stimulation in the absence of exogenously added TGFβ [20, 34, 49], the involvement of signaling cascades that regulate the gene expression of *SOX9* is limited.

**Fig. 1:**
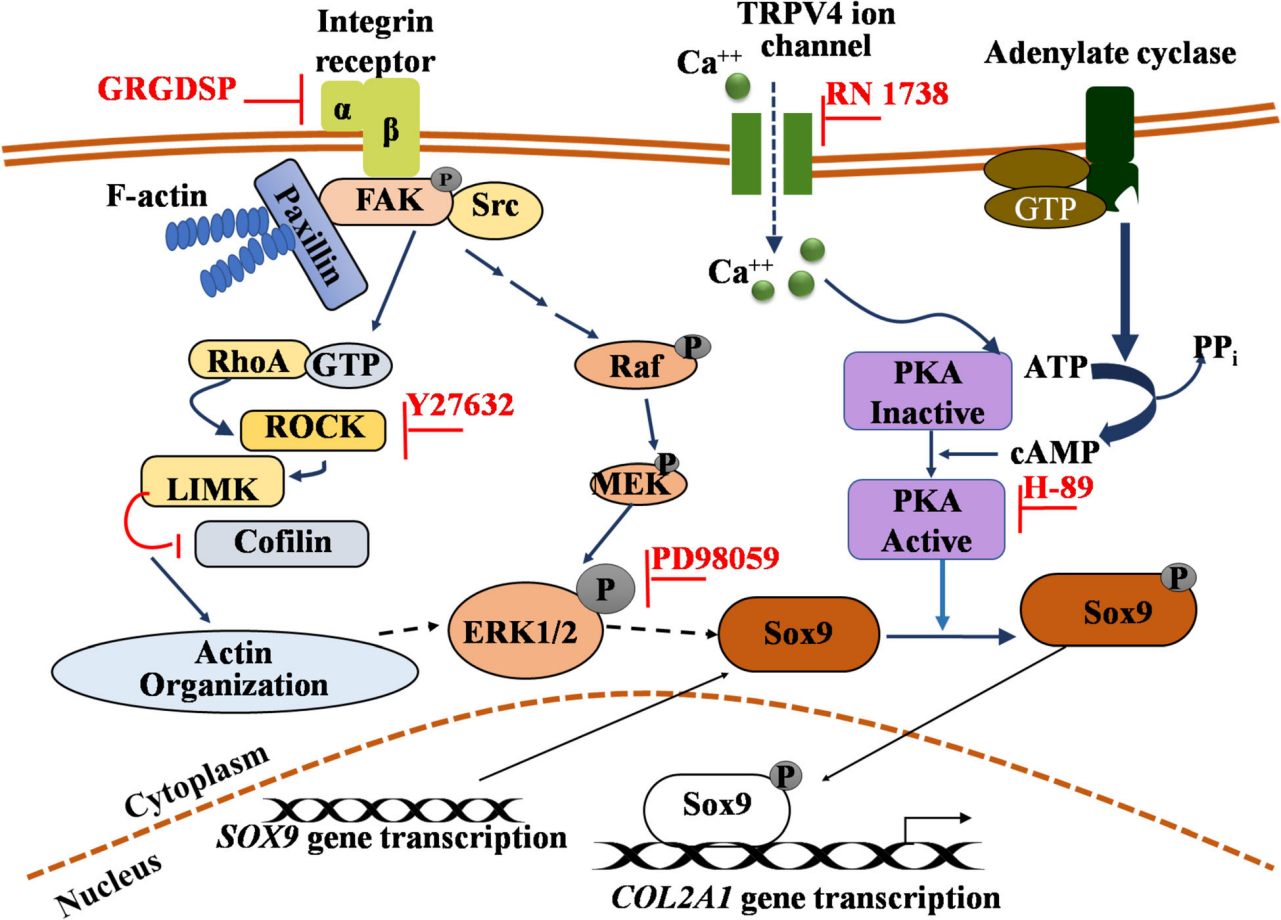
Schematic representation of the possible SOX9-directed pathways under cLIUS stimulation. The diagram shows putative signaling pathways that may be activated upon cLIUS stimulation in MSCs leading to the gene expression of *SOX9* and its target gene *COL2A1*. Select intracellular signaling molecules (ERK1/2 and PKA), upstream effectors (integrin and TRPV4), and the actin cytoskeleton were targeted by pharmacological inhibition to investigate the involvement of integrin-MAPK or calcium signaling through TRPV4 pathways leading to *SOX9* upregulation under cLIUS in MSCs

Long-term culture of scaffold-seeded with MSCs under cLIUS at 5 MHz, yielded a sustained and elevated expression of collagen II and glycosaminoglycan (GAG), notably in the absence of exogenously added growth factor TGFβ [38]. Our collective data indicated that cLIUS was chondroinductive as evidenced by the expression of cartilage-specific markers, notably *SOX9* [41, 40, 58, 73]. Thus, gaining a better understanding of the molecular events involved in the regulation of the expression of *SOX9* in MSCs under cLIUS can help develop preconditioning protocols based on cLIUS. We postulate that cell-surface receptors (integrin, TRPV4), key intracellular signaling molecules (ERK1/2, PKA), and the actin cytoskeleton are involved in transducing cLIUS stimulus leading to the expression of *SOX9* in MSCs and are schematically illustrated in Fig. 1. To our knowledge, this is the first study that seeks to elucidate the underlying signaling events that regulate *SOX9* upon cLIUS stimulation in MSCs. Thus, the assessment of select signaling molecules that are involved in regulating SOX9 under cLIUS was undertaken. Specificity was established using small molecule inhibitors of select signaling molecules. The study was motivated by the potential of cLIUS, a clinically translatable mode of stimulation in cartilage regeneration and rehabilitation.

## Methods

### 2D culture of MSCs

Human MSCs were purchased from Lonza (PT-2501, Walkersville, MD, USA) and expanded in alpha-Minimum Essential Medium (α-MEM) supplemented with 10% MSC-qualified fetal bovine serum (FBS) (Gibco, USA), 1× Glutamax (Gibco, USA), and 1× antibiotic-antimycotic solution (Gibco, USA) in a CO_2_ incubator at 37 °C, 5% carbon dioxide, and 99% humidity. MSCs harvested from passage 4–5 were employed in all experiments. MSCs were plated in 12-well TCP at following seeding densities: 1 × 10^5^ cells/well (RNA and protein extractions following treatment with inhibitors and non-treated controls), 1 × 10^4^ cells/well (for immunofluorescence studies following treatment with inhibitors and non-treated controls), 2 × 10^4^ cells/well (for RNA decay assay). All treatments with inhibitors and/or cLIUS were conducted after 24 h of initial seeding of MSCs in TCP.

### Treatment with inhibitors

MSCs were cultured in 12-well TCPs at 1 × 10^5^ seeding density per well (for RNA and protein extraction) with α-MEM medium supplemented with 10% MSC-qualified FBS, 1× Glutamax (Gibco, USA), and 1× antibiotic-antimycotic solution. For immunofluorescence staining, the 1 × 10^4^ cells per well were seeded onto sterilized 15-mm coverslips placed at the bottom of each well of a 12-well TCP. The MSCs were serum-starved prior to treatment with inhibitors by removing the growth medium containing 10% MSC-qualified FBS and replacing it with low serum medium containing 1% MSC-qualified FBS overnight. The serum-starved cells were incubated for 4 h in medium containing inhibitors solubilized in DMSO: 100 μg/ml GRGDSP (Sigma, USA), 50 μM PD98059 (Cell Signaling Technology, USA), 30 μM RN1738 (Tocris, USA), and 10 μM Y-27632 (Cell Signaling Technology, USA) separately for the inhibition of integrin, MEK/ERK1/2, and ROCK respectively prior to cLIUS stimulation. Non-cLIUS-treated MSCs incubated in medium containing DMSO (0.125% v/v) served as controls.

### cLIUS treatment regimen of 2D MSC cultures

Non-focused immersion transducers (Panametrics V300, 12.7 mm diameters, Panametrix, Waltham, MA, USA) were used to apply cLIUS (< 30 mW/cm^2^) to MSCs plated in 12-well TCP. The volume of medium per well was maintained at 4 ml to ensure complete immersion of ultrasound transducers in the well for effective propagation of ultrasound waves. Care was taken to avoid contact of the transducer surface to the cell layer at the bottom of the TCP. For cLIUS frequency study, cLIUS was applied at 5 MHz (2.5 Vpp), 2 MHz (6 Vpp), or 8 MHz (9.5 Vpp) at constant pressure amplitude of 14 kPa one time for 5 min. For inhibitor studies and RNA decay assay, cLIUS was applied one time for 5 min at 5 MHz (2.5 Vpp) with constant pressure amplitude of 14 kPa and subjected to qRT-PCR. For inhibitor studies, western blotting and immunofluorescence staining were conducted in addition to qRT-PCR following cLIUS stimulation.

### Quantitative RT-PCR (qRT-PCR)

Total RNA was isolated after 1 h upon cLIUS stimulation of MSCs in 2D. MSCs that received no cLIUS stimulation served as controls. Briefly, the medium was removed and cells were washed with HBSS (Gibco, USA). Cells were then lysed by adding 100 μl of Trizol (Invitrogen, USA) per well. RNA was then extracted from the homogenized cell lysates using the RNeasy Mini Kit (Qiagen, USA) as per manufacturer’s protocol. Cell lysates pooled from two wells served as one replicate. Three such replicates from three independent experiments were used for analysis (*n* = 3). qRT-PCR was carried out in Realplex™ real-time PCR system (Eppendorf, USA) using TaqMan® RNA-to-CT™ 1-Step Kit (Life Technologies, USA) as per the manufacturer’s guidelines. TaqMan® probes (Life Technologies, USA) for *SOX9* (Hs00165814_m1) was used to quantify mRNA expression of *SOX9*. The expression of mRNA transcripts was normalized to the housekeeping gene, *GAPDH* (Hs02786624_g1); expression and relative expression levels were calculated using the 2^−ΔΔCt^ method.

### Protein isolation and western blotting

Following treatment with inhibitors and/or cLIUS stimulation in 2D MSC cultures, total protein was isolated within 15 min of cLIUS stimulation (*n* = 3 biological replicates per group) as phosphorylation events are transient in nature. MSCs that received no cLIUS stimulation in the presence or absence of inhibitors served as respective controls. Briefly, the medium was removed, and cells were rinsed thoroughly in HBSS (Gibco, USA). The cells were lysed by adding 100 μl of Pierce IP lysis buffer (Thermo Scientific, Rockford, IL, USA) supplemented with 1× Halt protease and phosphatase inhibitor cocktail and 1X EDTA (Thermo Scientific, Rockford, IL, USA) per well. The cell lysates were centrifuged at 13,000*g* for 15 min, and the supernatant was used for protein quantification by standard BCA assay (QuantiPro™ BCA Assay Kit, Sigma-Aldrich, USA) as per the manufacturer’s guidelines. SDS-PAGE was conducted using NuPAGE gels (Invitrogen, USA) per the manufacturer’s instructions. Briefly, 20 μg protein in NuPAGE 4X lithium dodecyl sulfate sample buffer and NuPAGE 10× sample reducing agent was denatured at 75 °C for 15 min and loaded onto wells of 4–12% NuPAGE bis-tris gels, and electrophoresis was carried out. Proteins separated by SDS-PAGE were then transferred onto the PVDF membrane. Membranes were then blocked with 2% goat serum in 1× TBST (tris buffer saline with 0.1% tween20) for 2 h and incubated separately with primary antibodies: rabbit anti-phospho-p44/42 MAPK (ERK1/2) and p44/42 MAPK (ERK1/2) (catalog numbers 4377 and 4695, Cell Signaling Technology, USA) at a dilution of 1:1000 overnight at 4 °C. β-actin (catalog number 8H10D10, Cell Signaling Technology) or α-tubulin (catalog number 2144S, Cell Signaling Technology) was used as a loading control at a dilution of 1:5000. Following copious washing in TBST, the blots were incubated with HRP conjugated anti-rabbit IgG (1:5000 dilution, catalog number A0525, Sigma-Aldrich, USA) or anti-mouse IgG (1:10000 dilution, catalog number 12-349, Millipore-Sigma) for 2 h at room temperature. The blots were washed in TBST and then visualized by incubating with Clarity™ western ECL kit (Bio-Rad, USA) as per manufacturer’s instructions, finally captured with hyperfilm™ ECL (GE Healthcare Amersham™, USA). The blots were quantified by ImageJ™ software.

### Immunofluorescence staining

MSCs were seeded at a density of 1 × 10^4^ cells per coverslip per well in 12-well TCP. Immediately after treatment with inhibitors with or without cLIUS stimulation (5 min), MSCs were fixed in 4% paraformaldehyde for 20 min (*n* = 3). The cells were then rinsed in PBS and blocked with 2% goat serum in 1× TBST for 2 h. The blocking solution was removed, and the cells were incubated with a primary monoclonal antibody raised in rabbit against SOX9 (1:200 dilution, Catalog number 82630, Cell Signaling Technology, USA) at 4 °C overnight. After primary antibody incubation, the cells were washed with 1× TBST and incubated for 2 h at room temperature with goat anti-rabbit IgG conjugated with Alexa Fluor 488 (1:50 dilution, catalog number ab150077, Abcam, USA) followed by incubation with phalloidin-Alexa Fluor 594 (1:50 dilution, catalog number A12381, Molecular Probes, USA) for 30 min and staining with 300 nM DAPI (D1306, Molecular Probes, USA) for 5 min. The coverslips were rinsed and mounted onto glass slides with Prolong™ Gold antifade mountant (Invitrogen, USA) and viewed under a confocal microscope (Olympus IX81) at a magnification of ×60 (*z* step size = 5 μm). The fluorescence staining intensity in the images (*n* = 10–20 per group) from three independent experiments was quantified by measuring the integrated density of the cells using ImageJ software (NIH, Bethesda, USA). Quantification of actin filament number and length were measured by FilaQuant software (the University of Rostock, Institute of Mathematics, Mathematical Optimization) per user guidelines.

### Intracellular Ca^++^staining

MSCs were plated at an initial seeding density of 2 × 10^5^ cells/well. MSCs were pre-treated with the Fluo-4-AM probe (Catalog number F14201, Thermo Fisher Scientific, USA) at a concentration of 3 μM/ml in recording medium (20 mM HEPES, 115 mM NaCl, 5.4 mM KCl, 0.8 mM MgCl_2_, 1.8 mM CaCl_2_, 13.8 mM glucose) for 20 min, after which the medium was replaced with recording medium without Fluo-4-AM. Intracellular calcium was visualized 5 min after cLIUS stimulation (5 MHz. 2.5 Vpp, 5 min) under a fluorescence microscope at × 5 magnification (*n* = 3). Non-cLIUS-stimulated MSCs served as controls (*n* = 3).

### RNA decay assay

MSCs were cultured in 12-well TCP at a seeding density of 2 × 10^4^ cells per well in DMEM supplemented with 10% FBS, 1× antibiotic-antimycotic solution, and 25 μg/ml ascorbic acid. For RNA decay study, medium containing FBS was removed and replaced with a serum-free medium. cLIUS was then applied to the wells at 5 MHz and 2.5 Vpp for 15 min (*n* = 3). After cLIUS stimulation, the medium was immediately removed and replaced with serum-free DMEM containing 5 μg/ml actinomycin D (Cell Signaling Technology, USA). At each time point of 0 h, 0.5 h, 1 h, and 2 h post-actinomycin D treatment, the medium was removed and followed by total RNA extraction and qRT-PCR. Non-cLIUS-treated samples served as controls. Parallelly, MSCs were stimulated with cLIUS at 5 MHz and 2.5 Vpp for 15 min without subsequent actinomycin D treatment, and RNA was extracted at the aforementioned time points and subjected to qRT-PCR for measuring the relative amount of *SOX9* mRNA transcripts in the presence and absence of actinomycin D.

The ΔΔCt method is used for calculations. Ct values were first normalized to *GAPDH* and then normalized to the respective samples before the addition of actinomycin D, which were set to 1 [50]. This gives the decay of *SOX9* mRNA over time. To calculate the amount of *SOX9* mRNA transcripts at each time point, the Ct values (normalized to *GAPDH* first) of samples in the presence of actinomycin D were normalized to the Ct value of the sample in the absence of actinomycin D at respective time points.

### 3D encapsulation and culture of MSCs

MSCs were encapsulated in hyaluronan-based hydrogel using the HyStem-C hydrogel kit purchased from ESI-BIO (CA, USA) as per manufacturer’s instructions with slight modifications. Briefly, the hydrogel was prepared by mixing 2% Glycosil, 1% Gelin-S, and 4% Extralink solution supplied in the kit. Passage 4 MSCs were then harvested and resuspended in the hydrogel solution at an encapsulation density of 5 × 10^6^ cells/ml hydrogel. MSC-laden hydrogels were cast in sterile cylindrical inserts (4 mm height, 3 mm diameter) by pipetting 300 μl of the hydrogel-MSC mixture into the inserts. The inserts were then carefully removed after the solidification of MSC-laden hydrogels which typically ensued within (two constructs per well) were cultured in six-well tissue culture plates (TCP) in DMEM-high glucose medium supplemented with 10% fetal bovine serum (FBS) (Gibco, USA), 100 nM dexamethasone, 50 μg/ml ascorbic acid, and 1× antibiotic-antimycotic solution (Gibco, USA) at 37 °C and 5% CO_2_ with or without cLIUS for 6 weeks.

### cLIUS treatment regimen of 3D MSC constructs

Six-well TCPs containing MSC-hydrogel constructs were placed in plate holders of a cLIUS-assisted incubator developed at the Department of Chemical Engineering, University of Nebraska-Lincoln (UNL), USA, with operating procedures detailed elsewhere [51]. cLIUS was applied to the plates at 5 MHz frequency and 2.5 Vpp (14 kPa) for 20 min per application at four applications/day for a period of 6 weeks. At the end of 6 weeks, the constructs (*n* = 3) were fixed in 10% neutral buffered saline prior to immunohistochemistry. Non-cLIUS-treated constructs served as controls and were cultured in a CO_2_ incubator (37 °C and 5% CO_2_).

### Statistical analyses

The data are expressed as average ± standard deviation where the average is calculated from three independent experiments conducted in triplicate (*n* = 3). For qRT-PCR, ImageJ™, and FilaQuant data, Welch’s test was used for pair-wise comparisons of cLIUS-stimulated and non-cLIUS-stimulated control groups. For western blotting data and RNA decay assay, one-way analysis of variance (ANOVA) followed by post hoc Tukey’s test was employed for pair-wise comparisons. Statistically significant data (*α* = 0.5) was indicated by *p* values.

## Results

### cLIUS upregulates load-inducible genes

**Fig. 2:**
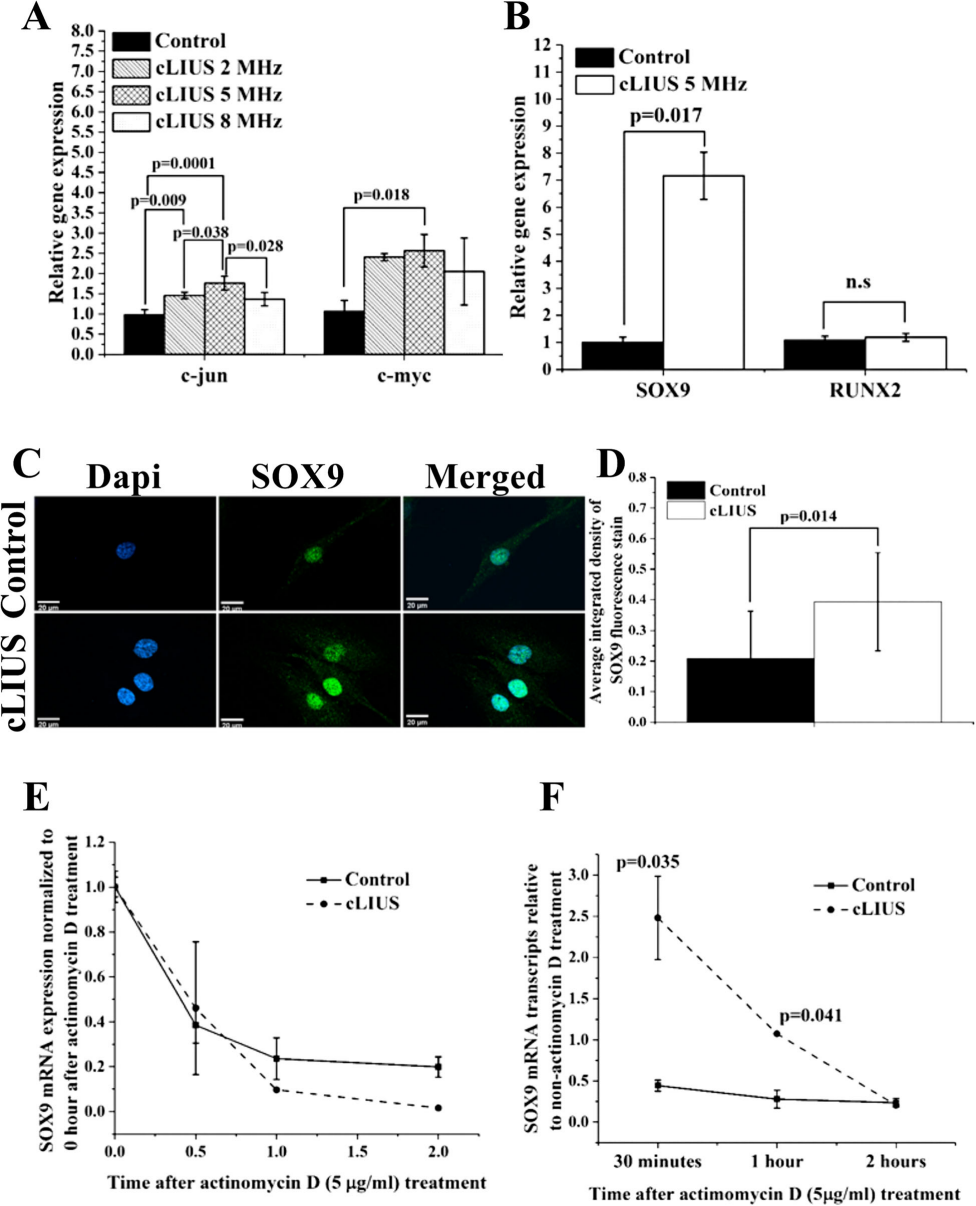
Effect of cLIUS on select genes, SOX9 localization, and SOX9 mRNA stability. Gene expression of **a** *c-MYC* and *c-JUN* and **b** *SOX9* and *RUNX2* in MSCs exposed to cLIUS at indicated frequencies were estimated by qRT-PCR (*n* = 3). MSCs plated at 1 × 10^5^ cells/well were exposed to cLIUS (14 kPa) at frequencies: 5 MHz (2.5 Vpp), 2 MHz (6 Vpp), or 8 MHz (9.5 Vpp) at constant pressure amplitude of 14 kPa one time for 5 min. Non-cLIUS-stimulated MSCs served as controls (*n* = 3). Data are shown as the mean ± standard deviation (Welch’s *t*-test). **c** MSCs in coverslips (*n* = 3) at an initial seeding density of 1 × 10^5^ cells/well were stimulated with cLIUS application at 14 kPa (5 MHz, 2.5 Vpp) for 5 min and fixed in 4% PFA. Confocal micrographs (× 60 magnification) of immunofluorescent staining of SOX9 (green) shows the localization of SOX9 in the MSCs under cLIUS (Scale bar = 20 μm). Nucleus was stained by Dapi (blue) and **d** quantified by ImageJ (*n* = 10–20). Data are shown as the mean ± standard deviation. **e** MSCs (2 × 10^4^ cells per well) were treated with 5 μg/ml actinomycin D, followed by stimulation with cLIUS at 14 kPa (5 MHz, 2.5 Vpp), for 15 min (*n* = 3). Total RNA was collected after 0 min, 30 min, 1 h, and 2 h of actinomycin D treatment, and the fold change in mRNA transcripts of *SOX9* were quantified by qRT-PCR. In a parallel experiment without actinomycin D treatment, total RNA was collected at indicated time points following cLIUS stimulation, and the fold change in *SOX9* mRNA transcripts was quantified by qRT-PCR (*n* = 3). Non-cLIUS-stimulated samples served as controls at respective time points (*n* = 3). Data represent the average ± standard deviation of fold change in *SOX9* mRNA levels normalized to time point 0. **f** Graphical representation of the amount of *SOX9* mRNA transcripts in actinomycin D-treated MSCs with or without cLIUS stimulation normalized to non-actinomycin D-treated samples at respective time points

To establish the frequency dependence of MSCs under cLIUS, the expression of load-inducible genes *c-MYC* and *c-JUN* that are independent of new protein synthesis [52], was evaluated using methods as detailed elsewhere [37] and shown in Fig. 2a. The gene expression of *c-MYC* and *c-JUN* were maximized at 5 MHz. Thus, in the current study, cLIUS at 5 MHz was employed in all experiments.

To first establish the chondroinductive potential of cLIUS at 5 MHz, the gene expression of the chondrogenic marker *SOX9* and osteogenic marker *RUNX2* were evaluated in MSCs and shown in Fig. 2b. Under cLIUS, a sevenfold increase in the gene expression of *SOX9* was observed whereas the gene expression of *RUNX2* remained unaffected when compared to non-cLIUS-stimulated control. In a separate study, no significant difference in the expression of osteogenic markers (*RUNX2*, *COL1A1*) and adipogenic markers (*CEBPA*, *PPARγ*) in MSCs was observed following 10 days of cLIUS (5 MHz) stimulation whereas chondrogenic markers (*SOX9* and *COL2A1*) remained significantly elevated under cLIUS stimulation when compared to non-cLIUS-stimulated controls (Additional file 1: Figure S1).

The transcriptional activity of SOX9 is contingent upon its subsequent nuclear translocation [53]. Thus, to further corroborate the gene expression results, the effect of cLIUS on the protein expression of SOX9 in cultures of MSCs was evaluated by immunofluorescence staining and is shown in Fig. 2c, d. The fluorescence intensity of SOX9 stain was significantly elevated (*p* = 0.014) in cLIUS-stimulated MSCs when compared to non-cLIUS-stimulated controls (Fig. 2d) and was highly concentrated in the nuclear region (Fig. 2c). Our collective data show that cLIUS increases the expression of the *SOX9* gene and protein in MSCs.

### cLIUS enhances the biosynthesis of SOX9 without affecting SOX9 mRNA stability

To ascertain the impact of cLIUS on the biosynthesis of SOX9 or its mRNA stability, RNA decay assay in the presence of actinomycin D was performed in 2D cultures of MSCs and shown in Fig. 2e, f. The degradation profile of *SOX9* mRNA under cLIUS and non-cLIUS-stimulated control were similar (Fig. 2e), thus indicating that cLIUS had no discernible effect on the stability of *SOX9* mRNA. In contrast, with respect to appropriate controls, higher levels of *SOX9* mRNA transcripts were noted under cLIUS (Fig. 2f). The results demonstrated that cLIUS promoted the biosynthesis of *SOX9* mRNA without affecting its stability.

### Assessing the role of select signaling molecules in regulating SOX9 under cLIUS

**Fig. 3:**
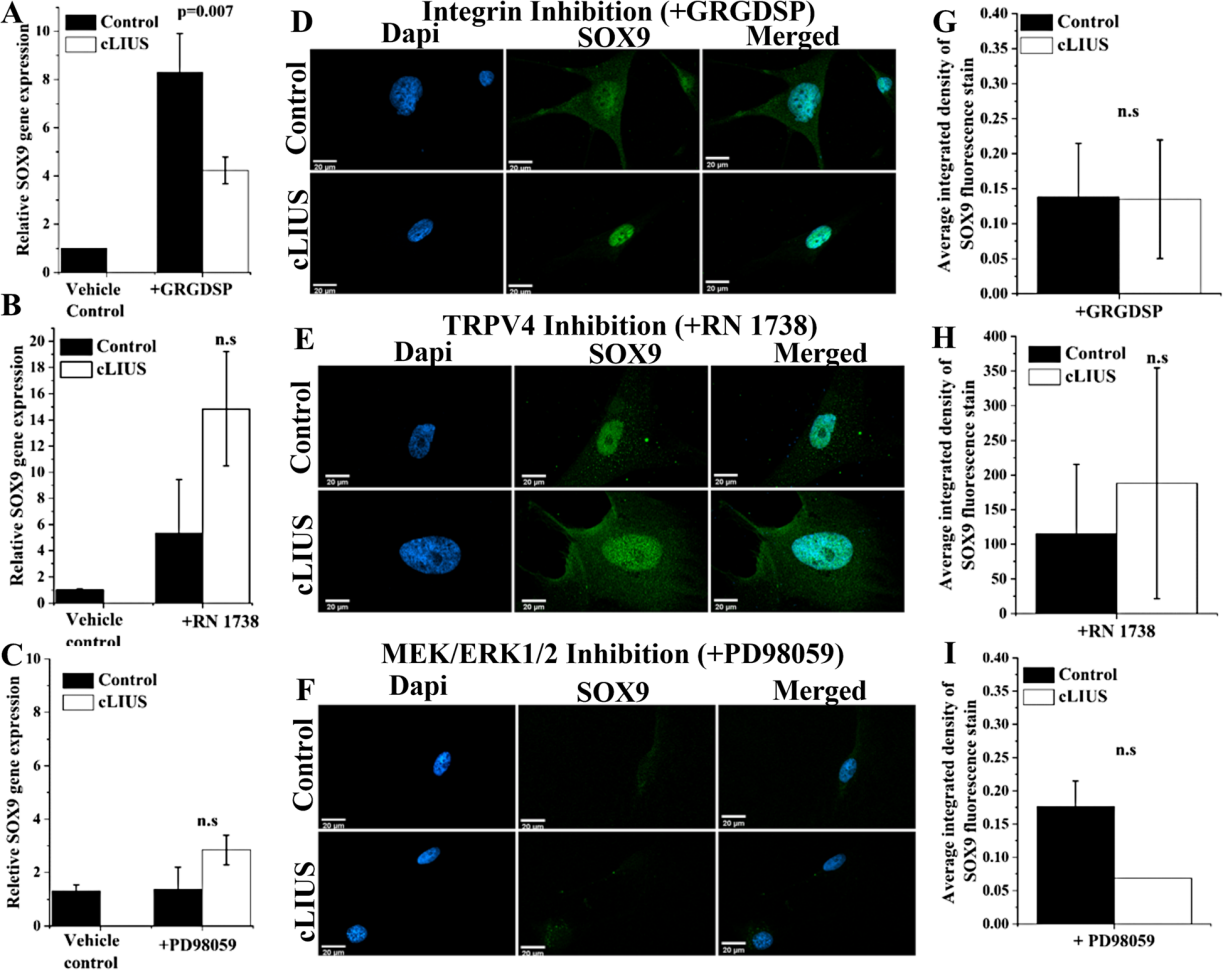
cLIUS-induced *SOX9* regulation under integrin, TRPV4, and MEK/ERK1/2 inhibition. *SOX9* gene expression in MSCs under **a** integrin inhibition by GRGDSP, **b** TRPV4 inhibition by RN 1738 (*n* = 3), and **c** MEK/ERK1/2 inhibition by PD98059 is shown. Serum-starved 2D cultures of MSCs were treated with inhibitors: 100 μg/ml GRGDSP (integrin inhibitor) or 30 μM RN1738 (TRPV4 inhibitor) for 4 h. Total RNA was collected 1 h after cLIUS stimulation at 14 kPa (5 MHz, 2.5 Vpp) for 5 min and the gene expression of *SOX9* was quantified by qRT-PCR. Non-cLIUS-stimulated MSCs incubated in DMSO served as vehicle controls (*n* = 3). Data represented as a mean ± standard deviation. **d–f** MSCs were grown in coverslips (*n* = 4–6 per treatment condition) at an initial seeding density of 1 × 10^5^ cells/well and were treated with inhibitors followed by the cLIUS application at 14 kPa (5 MHz, 2.5 Vpp) for 5 min and fixed in 4% PFA. Confocal micrographs (× 60 magnification) of immunofluorescent staining of SOX9 (green) shows the localization of SOX9 in the MSCs under **d** integrin inhibition by GRGDSP, **e** TRPV4 inhibition by RN1738, and **f** MEK/ERK1/2 inhibition by PD98059. The nucleus was stained with Dapi (blue). Scale bar represents 20 μm. **g–i** Quantification of SOX9 immunofluorescence intensity in control and cLIUS samples in the presence or absence of inhibitors by ImageJ (*n* = 10–20). Data are shown as the mean ± standard deviation of samples in triplicate. The *p* value represents the statistical significance, and n.s represents the non-significant difference as analyzed by Welch’s *t*-test

**Fig. 4:**
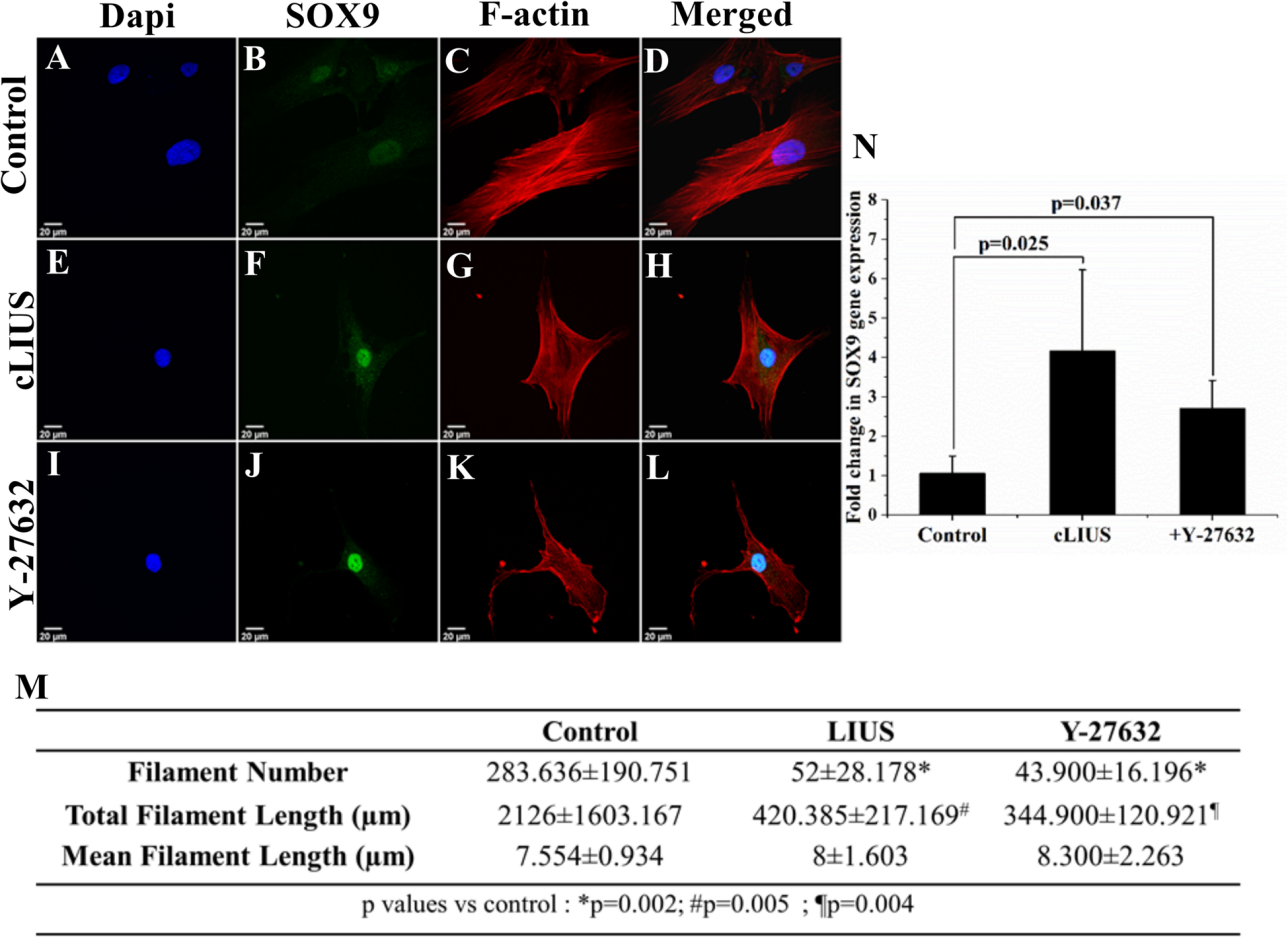
Effect of actin disruption on SOX9 upregulation. **a–l** Immunofluorescence staining of SOX9 (green) and F-actin filaments (red) in cLIUS-stimulated (5 MHz, 2.5 Vpp, 5 min, 1×) and Y-27632-treated MSCs grown on coverslips (*n* = 3) at an initial seeding density of 1 × 10^4^ cells/well (*n* = 3). Non-cLIUS-stimulated MSCs served as control (*n* = 3). Confocal micrographs were imaged at × 60 magnification. Scale bar represents 20 μm. **m** The gene expression of *SOX9* was evaluated by qRT-PCR in MSCs treated with Y-27632 or stimulated with cLIUS at 14 kPa (5 MHz, 2.5 Vpp) for 5 min (*n* = 3). Non-cLIUS-stimulated MSCs without Y-27632 treatment served as controls (*n* = 3). Data are shown as the mean ± standard deviation. Statistical significance was tested by Welch’s test. **n** Quantification of the actin filament number and length in non-cLIUS-stimulated control, cLIUS-, and Y-27632-treated cells (*n* = 10) by FilaQuant software

Studies have shown that key intracellular signaling molecules ERK1/2 [54] and PKA [48] are involved in the phosphorylation of SOX9. Upstream cell-surface effectors of ERK1/2 and PKA such as integrin and ion channel TRPV4 are known to be activated by mechanical stimuli [55–57]. Under cLIUS stimulation of MSCs, increased intracellular calcium ion levels were observed as measured by Fluo-4-AM assay as compared to non-cLIUS-stimulated control (Additional file 2: Figure S2). Therefore, the involvement of ion channel TRPV4 in the transduction of cLIUS signals to SOX9 gene expression was examined. To establish the specificity of cLIUS-induced upregulation of the *SOX9* gene, the gene expression and the nuclear localization of SOX9 under cLIUS were examined by qRT-PCR and immunofluorescence staining respectively, in the absence and presence of inhibitors of select signaling molecules in cultures of MSCs depicted in Fig. 1 and shown in Figs. 3 and 4.

### Inhibition of integrin or TRPV4 does not abrogate cLIUS-induced *SOX9* upregulation

In the absence of inhibitors, a sevenfold increase in the gene expression of *SOX9* was observed under cLIUS stimulation (*p* = 0.017) when compared to non-cLIUS-stimulated control (Fig. 2b). Upon inhibition of the cell-surface receptor integrin by GRGDSP, the gene expression of *SOX9* was significantly reduced but not completely abrogated when exposed to cLIUS (Fig. 3a). In the presence of RN 1738, an inhibitor of TRPV4, the gene expression of *SOX9* was upregulated; however, no difference in the expression levels was noted between cLIUS- and non-cLIUS-stimulated MSCs (Fig. 3b). Additionally, in the presence of inhibitors of integrin and TRPV4, no significant difference in the fluorescence intensity of SOX9 was noticed between cLIUS-stimulated and non-cLIUS-stimulated controls (Fig. 3d, e, g, h).

### Pharmacological inhibition shows that ERK-1/2 and not PKA transduces cLIUS signal

Prevention of the phosphorylation of the intracellular signaling molecule, ERK1/2, by the inhibition of its immediate upstream effector MEK1/2 by PD98059, abrogated the gene expression of *SOX9* (Fig. 3c). In the presence of H-89, an inhibitor of PKA, the gene expression of *SOX9* was upregulated in both cLIUS-stimulated MSCs and non-cLIUS-stimulated controls; however, no significant difference was observed between the groups (Additional file 2: Figure S2). Under MEK/ERK1/2 inhibition by PD98059, no nuclear localization of SOX9 was observed upon immunofluorescence staining in cLIUS-stimulated MSCs and non-cLIUS-stimulated controls (Fig. 3f, i). This result correlates with the previous finding that the gene expression of *SOX9* was abrogated upon MEK/ERK1/2 inhibition (Fig. 3c), thus confirming that the cLIUS-induced SOX9 expression was dependent on the phosphorylation of ERK1/2.

### The actin cytoskeleton is reorganized under cLIUS

Actin reorganization has been connected to *SOX9* upregulation [42, 45] where actin depolymerization correlated with the elevated gene expression of *SOX9* in cells treated Y-27632, an actin-depolymerizing compound [45]. Reorganization of actin in chondrocytes exposed to a similar cLIUS regimen has been previously reported [58]. Therefore, the actin cytoskeleton was examined in cultures of MSCs under cLIUS and in the presence of Y-27632 and shown in Fig. 4. Actin-phalloidin staining of MSCs depicted intact actin stress fibers in non-cLIUS-stimulated controls (Fig. 4c) while diffused or disrupted actin fibers were observed in MSCs exposed to cLIUS (Fig. 4g). Quantification of the number of actin fibers as measured by FilaQuant software (Fig. 4n) revealed a significant decrease (*p* = 0.002) in the number of actin filaments per cell from 283.64 ± 190.75 in non-cLIUS-stimulated controls to 52.00 ± 28.18 under cLIUS in MSCs. Similarly, diffused actin fibers were observed in Y-27632-treated MSCs (Fig. 4k) with a significant reduction in the actin filament number and length (Fig. 4n). The cLIUS-induced changes in the actin cytoskeleton were reversed within 24 h of withdrawal of cLIUS stimulus (data not included). In MSCs treated with Y-27632, increased expression of the *SOX9* gene (Fig. 4m) and the nuclear localization of the SOX9 protein (Fig. 4i, j) was noted. Similar trends were observed in MSCs exposed to cLIUS, which also exhibited actin disruption (Fig. 4j, e–h).

### ERK1/2 is phosphorylated under cLIUS

**Fig. 5:**
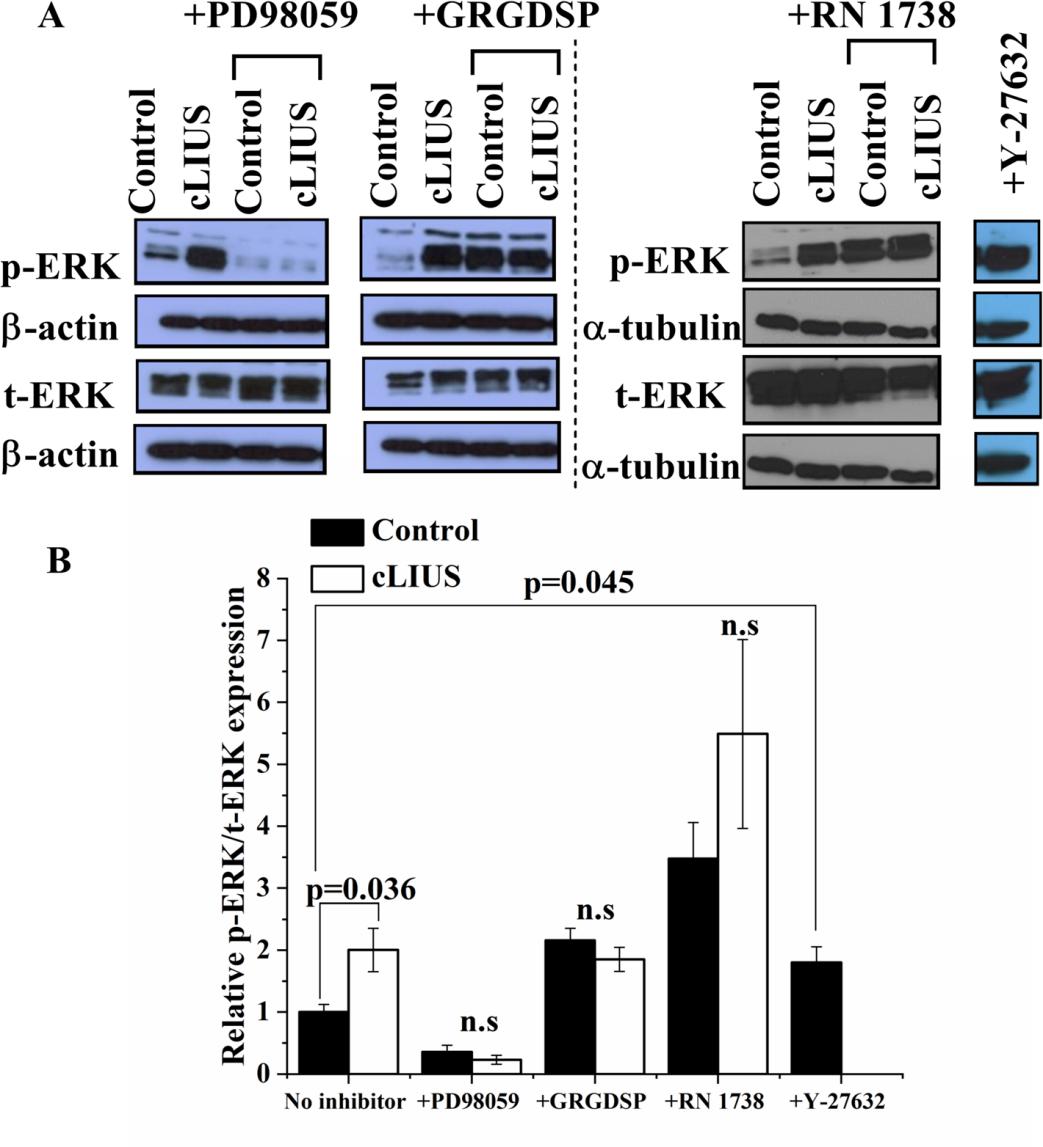
Phosphorylation of ERK1/2 under cLIUS. **a** Western blots depicting protein expression of phosphorylated ERK1/2 (p-ERK) and total ERK1/2 (t-ERK). 2D cultures of MSCs were grown in the presence or absence of inhibitors of MEK/ERK1/2 (PD98059), integrin (GRGDSP), or TRPV4 (RN1734) separately (*n* = 3), followed by exposure to cLIUS at 14 kPa (5 MHz, 2.5 Vpp) for 5 min. MSCs were lysed, total protein was extracted, and western blots were developed after SDS-PAGE. Non-cLIUS-stimulated MSCs served as respective controls (*n* = 3). **b** Quantification of bands observed in western blot by ImageJ (*n* = 3). Data are shown as the mean ± standard deviation of samples in triplicate. The *p* value represents a statistical significance and n.s. represents the non-significant difference (Welch’s test). Western blots were cropped for clarity and to exclude redundant adjacent lanes. Uncropped western blots are provided in supplementary files

Given the abolition of cLIUS-induced expression of SOX9 by inhibition of MEK/ERK1/2, the phosphorylation of ERK1/2 under cLIUS was investigated. The protein expression of phosphorylated ERK1/2 (p-ERK) and total ERK1/2 (t-ERK) under cLIUS in cultures of MSCs was analyzed by western blotting and shown in Fig. 5a. A twofold higher p-ERK/t-ERK ratio was noted under cLIUS stimulation as compared to non-cLIUS-stimulated control (Fig. 5b). Inhibition of MEK/ERK1/2 by PD98059 abrogated the phosphorylation of ERK1/2 in non-cLIUS-stimulated control as well as cLIUS-stimulated MSCs.

In contrast, inhibition of integrin and TRPV4 by GRGDSP and RN 1738, respectively, displayed increased levels of p-ERK/t-ERK ratio in both non-cLIUS-stimulated control and in cLIUS-stimulated MSCs. Thus, inhibition of the cell-surface receptors, integrin, and TRPV4, did not reduce the levels of p-ERK1/2, implying that other receptors of mechanical stimuli may be activated leading to the phosphorylation of ERK1/2 under cLIUS.

In MSCs treated with Y-27632 that promotes actin disruption, elevated levels of p-ERK were noted. Similar trends were observed in MSCs exposed to cLIUS which exhibited actin disruption (Fig. 4g) in concert with an increased p-ERK (Fig. 5a) and *SOX9* expression (Fig. 4e, f, m). Taken together, these results indicate a possible relationship between p-ERK and increased *SOX9* gene expression under cLIUS stimulation in MSCs.

### Chondrogenesis under cLIUS

**Fig. 6:**
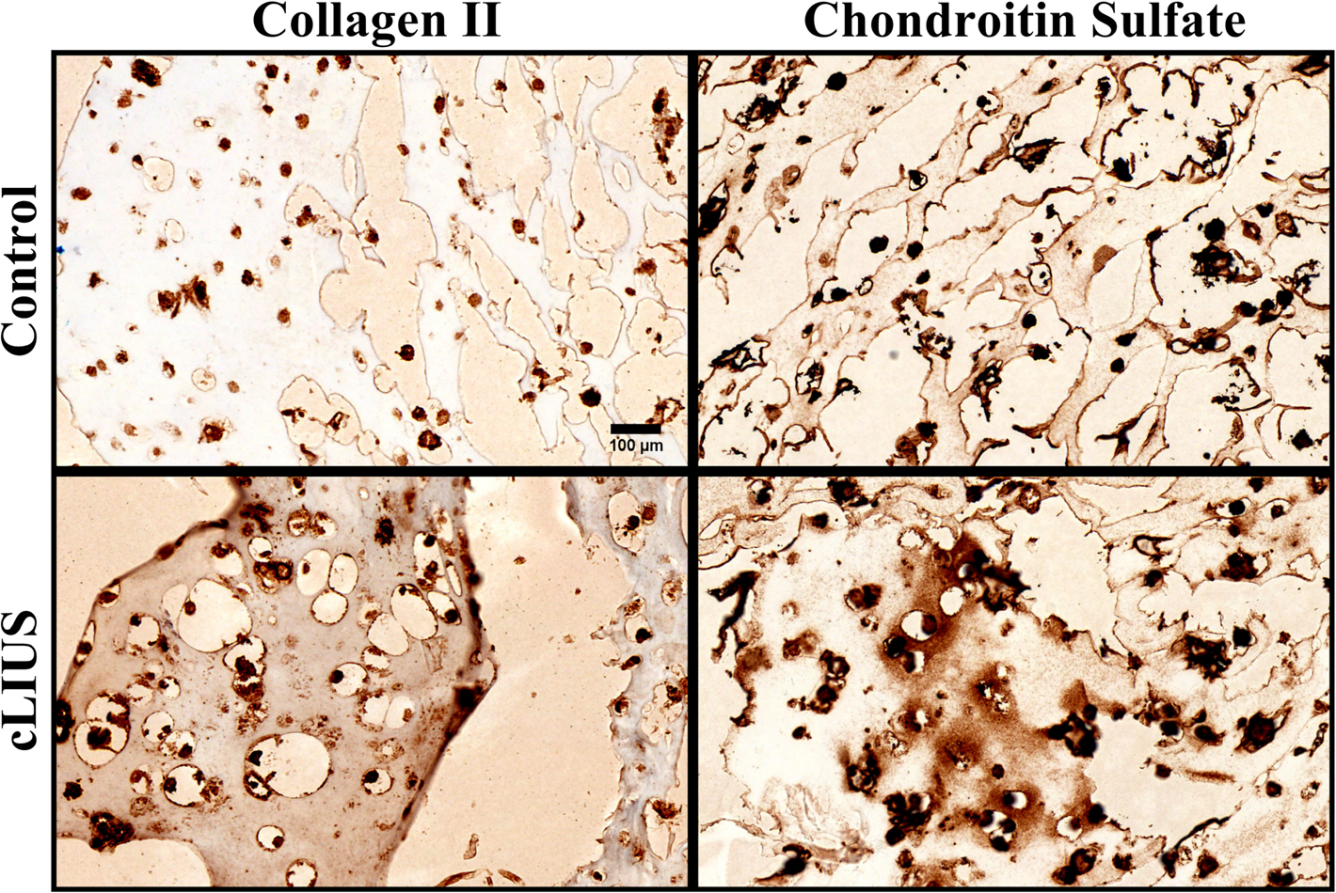
The culture of MSC-laden HyStem-C constructs under cLIUS stimulation. MSCs were encapsulated at a density of 5 × 10^6^ cells/ml of HyStem-C hydrogel and grown in DMEM medium supplemented with 10% FBS, 100 nM dexamethasone, and 50 μg/ml l-ascorbic acid for 6 weeks under cLIUS at 14 kPa (5 MHz, 2.5 Vpp), 20 min/application, and 4 applications/day (*n* = 3). Non-cLIUS-stimulated 3D constructs served as controls (*n* = 3). Representative images of 4-μm sections of the constructs stained immunohistochemically for collagen II and chondroitin sulfate is shown. Scale bar represents 100 μm

To examine the ability of cLIUS to induce a chondrogenic phenotype in MSCs in the absence of exogenously added TGFβ, MSC-laden-HyStem-C hydrogel constructs (3D) were cultured under cLIUS stimulation for 6 weeks and shown in Fig. 6. Immunohistochemical staining of HyStem-C hydrogel constructs displayed a higher deposition of collagen II and chondroitin sulfate in cLIUS-stimulated constructs as compared to non-cLIUS-stimulated constructs.

## Discussion

Chondrogenic differentiation of MSCs is predicated on the transcriptional activity *SOX9*, which is initiated by the direct binding of phosphorylated SOX9 to the target genes such as collagen type II, IX, XI, aggrecan, link protein; consequently, inducing their transcription [59, 60]. Therefore, priming of MSCs toward a chondrogenic lineage, by growth factors or mechanical stimuli, centers on the upregulation of *SOX9*. Given the increased levels of SOX9 protein and mRNA in MSCs under cLIUS stimulation [24, 38], the aim of the present study was to evaluate the underlying molecular events that regulate the cLIUS-induced expression of *SOX9*. Our results indicated that nuclear localization of the SOX9 protein, phosphorylated ERK1/2, and disrupted actin filaments were differentially regulated under cLIUS and play a role in transducing cLIUS signals to induce *SOX9* expression in MSCs.

The nuclear import of the transcription factor is related to its subsequent transcriptional activity [61]. For *SOX9* to be transcriptionally active, nuclear localization of SOX9 is vital for its target DNA binding [61–63]. Indeed, the most prominent inducer of chondrogenesis, TGFβ, operates by essentially increasing the transcriptional activity of the SOX9 protein through its stabilization and binding to the *COL2A1* gene via canonical or non-canonical pathways [43, 64]. In the present study, enhanced nuclear accumulation of SOX9 under cLIUS in MSCs (Fig. 2c) was indicative of its increased transcriptional activity as evidenced by elevated *COL2A1* and *SOX9* gene expression in MSCs (Additional file 1: Figure S1).

Mechanical stimuli including cLIUS have been previously documented to increase p-ERK1/2 and SOX9 [11, 65, 66] in both chondrocytes and MSCs [54]. However, a relationship between elevated levels of p-ERK and the gene expression of *SOX9* was not established. Our results demonstrate that under cLIUS, *SOX9* upregulation in MSCs was dependent on the phosphorylation of ERK1/2 and the specificity was established using MEK/ERK1/2 inhibitor. Similar results were observed in FGF-2 treated immortalized ADTC5 cell lines [67]. The exact molecular mechanism of p-ERK1/2 directed regulation of *SOX9* expression remains to be investigated.

In the present study, cLIUS was shown to disrupt actin filaments in MSCs and a correlation between the perturbed actin cytoskeleton and *SOX9* upregulation under cLIUS was inferred. Interestingly, reorganization of the actin cytoskeleton was noted as a precondition to differentiation [68]. Previous work alludes to the role of actin disrupted by the cytoskeletal drug, Y-27632, in inducing chondrogenesis in limb mesenchymal cultures by elevating the gene expression of *SOX9* [44, 45]. In our study, cLIUS-induced disruption of the actin filaments was concomitant with increased *SOX9* gene expression in MSCs (Fig. 4). To establish specificity, actin stabilizer jasplakinolide was added; however, the results were inconclusive. In our study, the gene expression of *SOX9* was dependent on ERK1/2 phosphorylation. We postulate that increases in *SOX9* observed under cLIUS or Y-27632 as a result of actin disruption was influenced by the increased levels of p-ERK (Fig. 5). The treatment of non-stimulated MSCs by the MEK/ERK1/2 inhibitor, which prevented the phosphorylation of ERK1/2, disrupted actin but did not result in the upregulation of *SOX9* as expected (Fig. 3, Additional file 3: Figure S3). Thus, our present study indicated that the phosphorylation of ERK1/2 was the key molecular event impacting the expression of *SOX9*.

As the phosphorylation of ERK1/2 was pivotal in the upregulation of *SOX9* under cLIUS, the role of upstream effectors of ERK1/2 was evaluated to understand the pathways through which cLIUS signals were transduced to ERK1/2, ultimately leading to the gene expression of *SOX9* in MSCs. The involvement of the integrin-mediated MAPK pathway in transducing cLIUS signals to ERK1/2 in chondrocytes has been reported [65, 69]. In another study, pLIUS was shown to immediately elevate levels of p-ERK1/2 [27] through the activation of TRPV4-mediated calcium signaling pathway in chondrocytes. In the current study, individual blockage of either integrin or TRPV4 failed to abrogate p-ERK1/2 under cLIUS stimulation in MSCs, and hence, *SOX9* remained elevated. Thus, other sensors of mechanical signals that also regulate the phosphorylation of ERK1/2 could play a role in the ERK1/2-mediated *SOX9* upregulation under cLIUS in MSCs.

The main purpose of the study was to evaluate the chondroinductive ability of cLIUS by monitoring the gene and protein expression of SOX9, transcription factor that controls the expression of collagen II (*COL2A1*). Thus, the focus was on the molecular consequences following cLIUS, which are best evaluated upon a single exposure to LIUS. To offer proof of the relevance of these molecular events to chondrogenesis, MSC constructs were cultured in Hystem-C™ hydrogels under cLIUS (note no exogenous growth factors were added) for 6 weeks (Fig. 6). Increased deposition of collagen II and chondroitin sulfate at the end of the culture period demonstrated MSC chondrogenesis upon extended exposure to cLIUS. Previously, the long-term 3D culture of MSCs in scaffolds under cLIUS stimulation, notably in the absence of TGFβ, also yielded an elevated expression of collagen II and SOX9 protein [38]. Further, the biochemical, as well as mechanical properties (compressive strength and aggregate modulus), were significantly higher in MSC-laden scaffold constructs exposed to cLIUS stimulation for 8 weeks as opposed to non-cLIUS-stimulated controls [38]. Collectively, our results demonstrated the chondroinductive ability of cLIUS in 3D cultures of MSCs in the absence of TGFβ.

## Conclusions

In summary, our study identified the phosphorylation of ERK1/2, increased nuclear localization of the SOX9 protein, and disrupted actin as the events mediating increased *SOX9* gene expression under cLIUS. Most notably, cLIUS-induced upregulation of *SOX9* was dependent on the phosphorylation of ERK1/2, signifying the involvement of ERK1/2 signaling on the *SOX9* gene and protein expression. The involvement of endogenously secreted TGFβs in MSCs in response to cLIUS was excluded, as the current study focused on the immediate molecular events following one dose of cLIUS stimulation. However, the role of intrinsic TGFβ signaling in concert with cLIUS in MSCs will be investigated in future studies. The role of additional physical perturbations such as nuclear deformation and chromatin reorganization on MSCs in response to cLIUS will also be undertaken in future investigations. Additionally, the results presented in the current study remain to be verified in 3D cultures and in cartilage explants.

## Supplementary information

Additional file 1: **Figure S1.** Gene expression of select osteogenic, chondrogenic and adipogenic markers under cLIUS. MSCs were grown at an initial seeding density of 2 x 10^5^ cells/ml in 12-well TCP. MSCs were exposed to cLIUS at 14 kPa (5.0 MHz, 2.5 Vpp), 5 min/application, 4X/day for a period of 10. MSCs (*n*=3) were treated with Trizol and total RNA was extracted using RNeasy Mini Kit (Qiagen, USA) as per manufacturer’s protocol. Non-cLIUS-stimulated MSCs served as control (n=3). qRT-PCR was carried out in Realplex™ real-time PCR system (Eppendorf, USA) using TaqMan® RNA-to-CT™ 1-Step Kit (Life Technologies, USA) as per manufacturer’s guidelines. The gene expression of select osteogenic (Runx2, SPP-1, and COL1A1), chondrogenic (SOX9 and COL2A1) and adipogenic (CEBPA and PPARγ) was evaluated. Data represent mean ± standard deviation. *p-value <0.05 & compared with control.

Additional file 2: **Figure S2.** Intracellular Ca^++^ influx under cLIUS and SOX9 gene expression under PKA inhibition: a MSCs were plated at an initial seeding density of 2 X 10^5^ cells/well on 12-well TCP. MSCs were pre-treated with the Fluo-4-AM probe (3 μM) in a recording medium (20 mM HEPES, 115 mM NaCl, 5.4 mM KCl, 0.8 mM MgCl_2_, 1.8 mM CaCl_2_, 13.8mM glucose) for 20 minutes, after which the medium was replaced with recording medium without Fluo-4-AM. Intracellular calcium was visualized 5 minutes after cLIUS stimulation (5 MHz. 2.5 Vpp, 5 minutes) under a fluorescence microscope at 5X magnification(n=3). Non-cLIUS-stimulated MSCs served as controls (n=3). Phase-contrast images (5X magnification) depict the general morphology of the stained cells. b The gene expression of SOX9 in non-cLIUS-stimulated and cLIUS-stimulated MSCs in the presence of H-89, an inhibitor of PKA (20μg/ml). Total RNA was collected 1 hour after cLIUS treatment in MSCs treated with H-89 and subjected to qRT-PCR. Non-cLIUS stimulated MSCs served as control. Data represented as a mean ± standard deviation and normalized to vehicle control.

Additional file 3: **Figure S3.** Figure. Actin staining in MSCs under ERK1/2 inhibition. MSCs were treated with MEK/ERK1/2 inhibitor PD98059 for 4 h and fixed in 4% paraformaldehyde. Immunofluorescence staining for F-actin (red) by phalloidin-Alexa Fluor 594 was carried out and the representative confocal image is presented (*n* = 3). Scale bar represents 20 μm.

## Data Availability

All data generated or analyzed during this study are included in this published article [and its supplementary information files].

## Abbreviations

2D: 2-Dimensional
3D: 3-Dimensional
ACI: Autologous chondrocyte implantation
cLIUS: Continuous low-intensity ultrasound
DMSO: Dimethyl sulfoxide
ERK1/2: Extracellular signal-regulated kinase 1 and 2
FBS: Fetal bovine serum
GAG: Glycosaminoglycans
MACI: Matrix-assisted autologous chondrocyte implantation
MAPK: Mitogen-activated protein kinase
MSC: Mesenchymal stromal cell
PKA: Protein kinase A
ROCK: Rho kinase
SOX9: Sex determining region Y-box 9
TGFβ: Transforming growth factor β
TRPV4: Transient receptor potential vanilloids 4
α-MEM: α-Minimal essential medium
