# Supplementary figures and images for: Preconditioning of mesenchymal stromal cells with low-intensity ultrasound: influence on chondrogenesis and directed SOX9 signaling pathways
Source: Stem Cell Res Ther. 2020 Jan 3;11:6. doi: 10.1186/s13287-019-1532-2 (PMC6942392; doi:10.1186/s13287-019-1532-2)

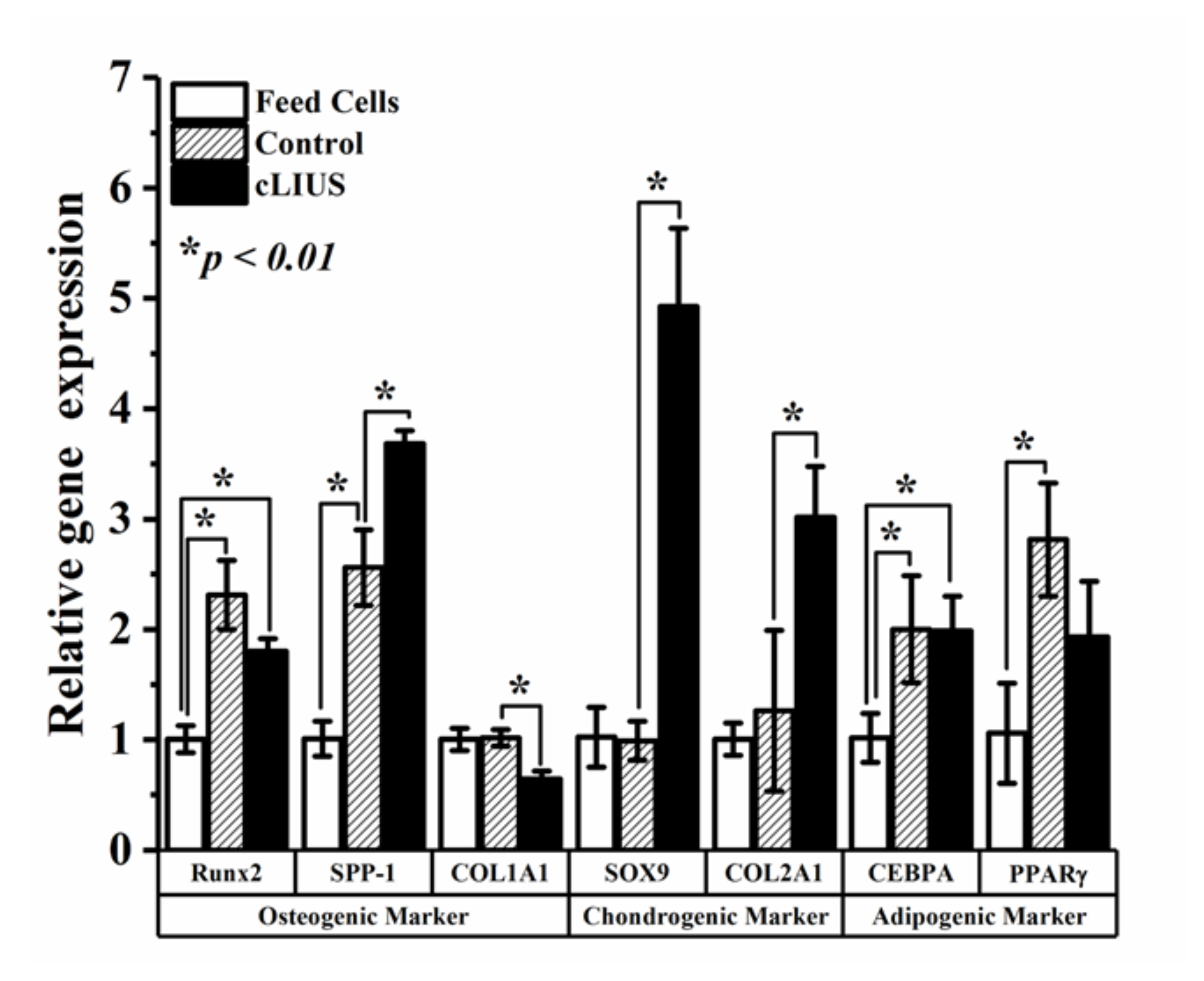

Supplement: Supplementary file 1 — Figure S1. Gene expression of select osteogenic, chondrogenic and adipogenic markers under cLIUS. MSCs were grown at an initial seeding density of 2 x 10^5^ cells/ml in 12-well TCP. MSCs were exposed to cLIUS at 14 kPa (5.0 MHz, 2.5 Vpp), 5 min/application, 4X/day for a period of 10. MSCs (n=3) were treated with Trizol and total RNA was extracted using RNeasy Mini Kit (Qiagen, USA) as per manufacturer’s protocol. Non-cLIUS-stimulated MSCs served as control (n=3). qRT-PCR was carried out in Realplex™ real-time PCR system (Eppendorf, USA) using TaqMan® RNA-to-CT™ 1-Step Kit (Life Technologies, USA) as per manufacturer’s guidelines. The gene expression of select osteogenic (Runx2, SPP-1, and COL1A1), chondrogenic (SOX9 and COL2A1) and adipogenic (CEBPA and PPARγ) was evaluated. Data represent mean ± standard deviation. *p-value <0.05 & compared with control. [file 13287_2019_1532_MOESM1_ESM.tif]

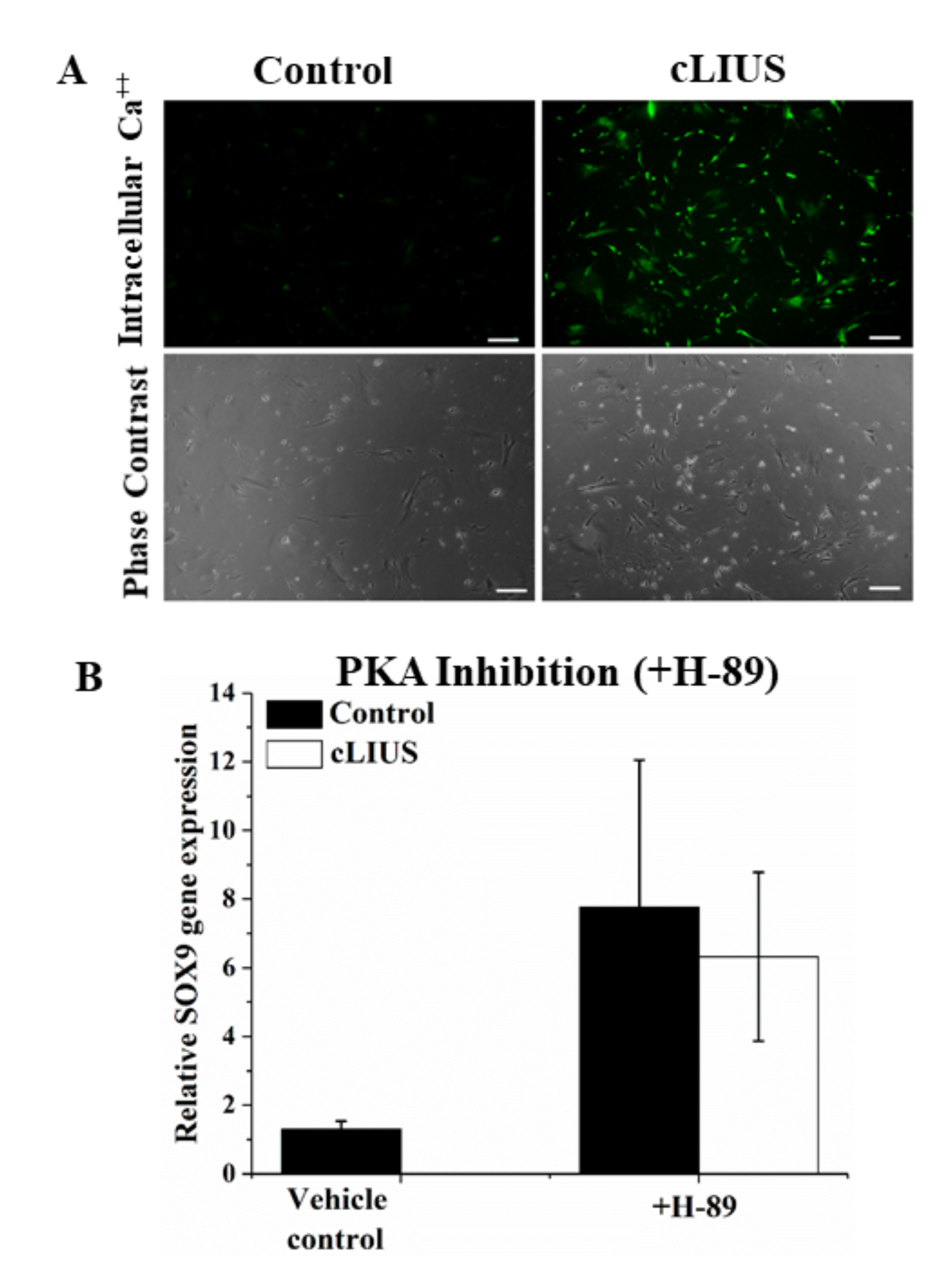

Supplement: Supplementary file 2 — Figure S2. Intracellular Ca++ influx under cLIUS and SOX9 gene expression under PKA inhibition: a MSCs were plated at an initial seeding density of 2 X 105 cells/well on 12-well TCP. MSCs were pre-treated with the Fluo-4-AM probe (3 μM) in a recording medium (20 mM HEPES, 115 mM NaCl, 5.4 mM KCl, 0.8 mM MgCl2, 1.8 mM CaCl2, 13.8mM glucose) for 20 minutes, after which the medium was replaced with recording medium without Fluo-4-AM. Intracellular calcium was visualized 5 minutes after cLIUS stimulation (5 MHz. 2.5 Vpp, 5 minutes) under a fluorescence microscope at 5X magnification(n=3). Non-cLIUS-stimulated MSCs served as controls (n=3). Phase-contrast images (5X magnification) depict the general morphology of the stained cells. b The gene expression of SOX9 in non-cLIUS-stimulated and cLIUS-stimulated MSCs in the presence of H-89, an inhibitor of PKA (20μg/ml). Total RNA was collected 1 hour after cLIUS treatment in MSCs treated with H-89 and subjected to qRT-PCR. Non-cLIUS stimulated MSCs served as control. Data represented as a mean ± standard deviation and normalized to vehicle control. [file 13287_2019_1532_MOESM2_ESM.tif]

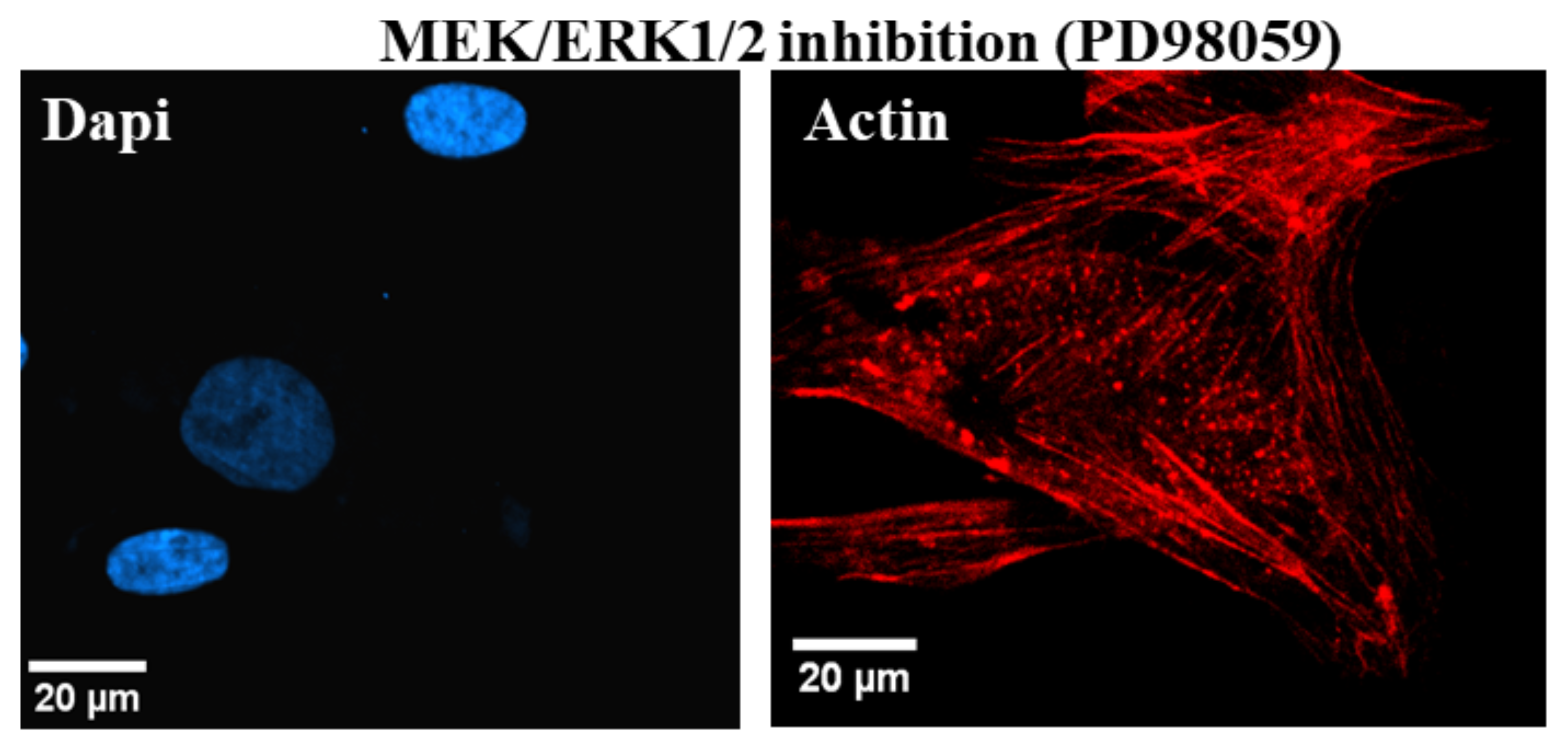

Supplement: Supplementary file 3 — Figure S3. Figure. Actin staining in MSCs under ERK1/2 inhibition. MSCs were treated with MEK/ERK1/2 inhibitor PD98059 for 4 h and fixed in 4% paraformaldehyde. Immunofluorescence staining for F-actin (red) by phalloidin-Alexa Fluor 594 was carried out and the representative confocal image is presented (n = 3). Scale bar represents 20 μm. [file 13287_2019_1532_MOESM3_ESM.tif]
